# Visualizing Social and Behavior Change due to the Outbreak of COVID-19 Using Mobile Phone Location Data

**DOI:** 10.1007/s00354-021-00139-x

**Published:** 2021-11-02

**Authors:** Takayuki Mizuno, Takaaki Ohnishi, Tsutomu Watanabe

**Affiliations:** 1 National Institute of Informatics, 2-1-2 Hitotsubashi, Chiyoda-ku, Tokyo, 101-8430 Japan; 2 The Canon Institute for Global Studies, 11th Floor, ShinMarunouchi Building, 5-1-1 Marunouchi, Chiyoda-ku, Tokyo, 100-6511 Japan; 3 Graduate School of Artificial Intelligence and Science, Rikkyo University, 3-34-1 Nishi-Ikebukuro, Toshima-ku, Tokyo, 171-8501 Japan; 4 Graduate School of Economics, University of Tokyo, 7-3-1 Hongo, Bunkyo-ku, Tokyo, 113-0033 Japan

**Keywords:** COVID-19, Social and behavior change (SBC), Mobile phone location, Stay-at-home

## Abstract

We visualize the rates of stay-home for residents by region using the difference between day-time and night-time populations to detect residential areas, and then observing the numbers of people leaving residential areas. There are issues with measuring stay-home rates by observing numbers of people visiting downtown areas, such as central urban shopping centers and major train stations. The first is that we cannot eliminate the possibility that people will avoid areas being observed and go to other areas. The second is that for people visiting downtown areas, we cannot know where they reside. These issues can be resolved if we quantify the degree of stay-home using the number of people leaving residential areas. There are significant differences in stay-home levels by region throughout Japan. By this visualization, residents of each region can see whether their level of stay-home is adequate or not, and this can provide incentive toward compliance suited to the residents of the region.

## Introduction

The coronavirus disease that began at end of 2019 in Wuhan, China, expanded around the world in the blink of an eye, and as of December 8, 2020, more than 67.47 million people around the world have been infected, and approximately 1.54 million have lost their lives. To stop the pandemic, regions around the world (Wuhan, Italy, India, Malaysia, New Zealand, Philippines, Singapore, UK, USA, Indonesia, France, Ireland, Australia and others) have invoked laws prohibiting outings. On April 7, the government of Japan declared a state of emergency in seven prefectural regions. To prevent infection by the coronavirus, Prime Minister Abe appealed for people to reduce their contact with others by at least 70%, and as much as 80% [1]. Then, on April 16, the state of emergency was expanded to cover all of Japan, and to continue till May 25 [2]. This included a strong request that citizens stay at home, and to not go out for any reason that was not urgent or necessary.

To visualize the stay-home levels for this request, the Japanese government has posted data on the cabinet secretariat’s web page for Novel Coronavirus Disease Countermeasures, regarding changes in the movements of the population based on location information from mobile phones and other sources [3]. Specifically, it shows rates of reduction in daytime populations compared with values during ordinary times in areas that generally have large influxes of people, such as around city-center areas and major train stations. However, there are issues with measuring levels of stay-home by observing conditions in areas with large influxes of people. First, the possibility that people will avoid the areas being observed and go out to other areas cannot be excluded. For example, although areas like Shibuya and Shinjuku had hardly a soul on the weekends, many of the local, smaller shopping areas were crowded. When peoples’ destinations change in this way, it is meaningless to use fixed observation points in normally busy areas. A second issue is that it is not clear where the residents who are not in compliance are from.

Since the government does not know the stay-home levels in each residential area, requests for stay-home compliance were made uniformly, not by region. For those in regions that are actively complying, the requests could seem excessive, while the requests could be inadequate in areas where residents are not complying adequately. It is also difficult for residents of a given region to know whether their level of stay-home is appropriate for the rates of infection in their area, and this could have undermined the incentive to comply for residents in some regions. Thus, it is necessary to quantify the rates at which residents in each region are complying with respect to staying home, and to show how this relates to controlling infection, so that the effects of measures against the disease can be verified.

Various methods have been studied for measuring peoples’ level of stay-home. Credit card transaction data have been used to measure compliance by comparing consumers’ expenses on eating out and other forms of entertainment with their normal patterns, according to residential area, sex, and age group [4]. Anonymized mobile phone location data have been used to measure when people are at home and how far they have traveled [5, 6]. Geo-tagged tweets have also been used to measure the reduction in traveling distances [7]. A correlation between changes in the amount of time spent at home, the workplace, and shopping, and rates of infection has been shown [8–10]. It has been shown that compliance rates for staying at home are not uniform across different regions [11]. Stagnation of economic activity with an increase in the number of infections has been observed based on night-time illumination and power consumption [12–14]. A decrease in the number of customers at shopping malls has also been observed in satellite images [15].

In this research, real-time hourly population distributions estimated from the approximately 80 million NTT DOCOMO mobile phones in Japan were used to observe the flow of people out of residential areas, rather than the flow into commercial areas. The location data were anonymized, so that the movements of individual mobile phones could not be observed. Observation data consisted of hourly population-distribution snapshots. By observing changes in population in residential areas that do not attract visitors for other reasons, where inflows and outflows consist of only local residents, we estimate the number of residents leaving each area. By comparing daily numbers of residents leaving residential areas with the average values from January, 2020, when the spread of the coronavirus was still small, the rates of stay-home by residents in each area are computed. In this way, changes in the rate of stay-home by region can be determined, and their relation to controlling infection can be studied statistically. Regions with low rates of stay-home compared to their infection conditions can then be identified, and effective requests for stay-home compliance can be made according to the actual conditions in each region.

“Population Flow Big Data” describes hourly human population distribution data for Japan, which was used to measure flows of people. In “Extracting Residential Areas”, day-time and night-time populations are used to extract residential areas. In “Definition of Stay-Home Rate”, the differences between night-time and day-time populations are used to estimate the number of people going out, and we propose a method for computing the rates of stay-home for residents of a region, by correcting for weekly cycles and comparing with values during ordinary times. In “Trends in Stay-Home Compliance Throughout Japan”, we discuss examples estimating rates of stay-home in Hokkaido on March 1, the weekend after a state of emergency was declared, and in all of Japan on March 3, during the “STAY HOME" week in Tokyo. We give time-series of stay-home rates in each region, and discuss their relationship with events that occurred and differences in stay-home rates between regions. We also show that the stay-home rate does not depend much on sex and age. In “Relationship Between the Stay-Home Rate and the Spread of Infection”, we investigate how the spread of infection was related with the extent to which people stay at home. Finally, “Conclusion” provides a conclusion and issues for further study.

## Population Flow Big Data

This research used real-time domestic population distribution statistics, called Mobile Space Statistics^®^ (“Mobaku”) provided by DOCOMO Insight Marketing, Inc [16]. Mobile Space Statistics consists of hourly, real-time population distributions in Japan, estimated using the approximately 80 million NTT DOCOMO mobile phones in the country. These population distributions are called population flows. Mobile phone base stations in each area periodically update the mobile phones that are in the area, and these data are augmented with NTT DOCOMO subscriber information including age (15–79 years old), sex, residence area (down to the city/town/village level), and NTT DOCOMO’s market share in the area, to estimate population flows on a 500 m grid for age groups from 15–79 years of age throughout Japan. Note that grid sections with low numbers of people are excluded to protect the privacy of individuals. The entire country was partitioned into 500 m squares, and population flows were recorded for each region, with the location, and according to age, sex, and residence (to the city/town/village level). Data for the dates from January 6 to June 22, 2020, were analyzed for this research.

## Extracting Residential Areas

The national census was used to examine usual-residence population distributions for all of Japan. Most people spend nights at this residence, so it is also called night-time population. During the day, most people leave their homes and go to school or work. We also compute this day-time population distribution by applying aggregate values of work and school locations from the census to the night-time population values. The ratio of day-time to night-time populations is called day-to-night population ratio, and this ratio has been reported to be 0.8 or less for urban bedroom communities, such as Miyamae Ward in Kawasaki City (0.74), Minami Ward in Saitama City (0.76), and Aoba Ward in Yokohama City (0.77) [17].

The national census is conducted once every five years, so it does not provide real-time day-time and night-time populations. Thus, the Statistics Bureau at the Ministry of Internal Affairs and Communications is conducting a study of whether mobile phone base station data and user GPS data collected by smartphone applications could be used to fill in spaces not covered by the five-year national census [18]. This study includes a comparison of day-time and night-time populations estimated from base-station and GPS data, from population flows in various areas of Tokyo from the same year, with the values from the census. The hourly population flow data showed clear 24-h cycles. However, the relative population flows for each area, comparing night-time values from midnight to 6 am with morning-to-evening values from 9 am to 6 pm, were stable, with the former being close to the night-time population, and the latter being close to the day-time population.

**Fig. 1:**
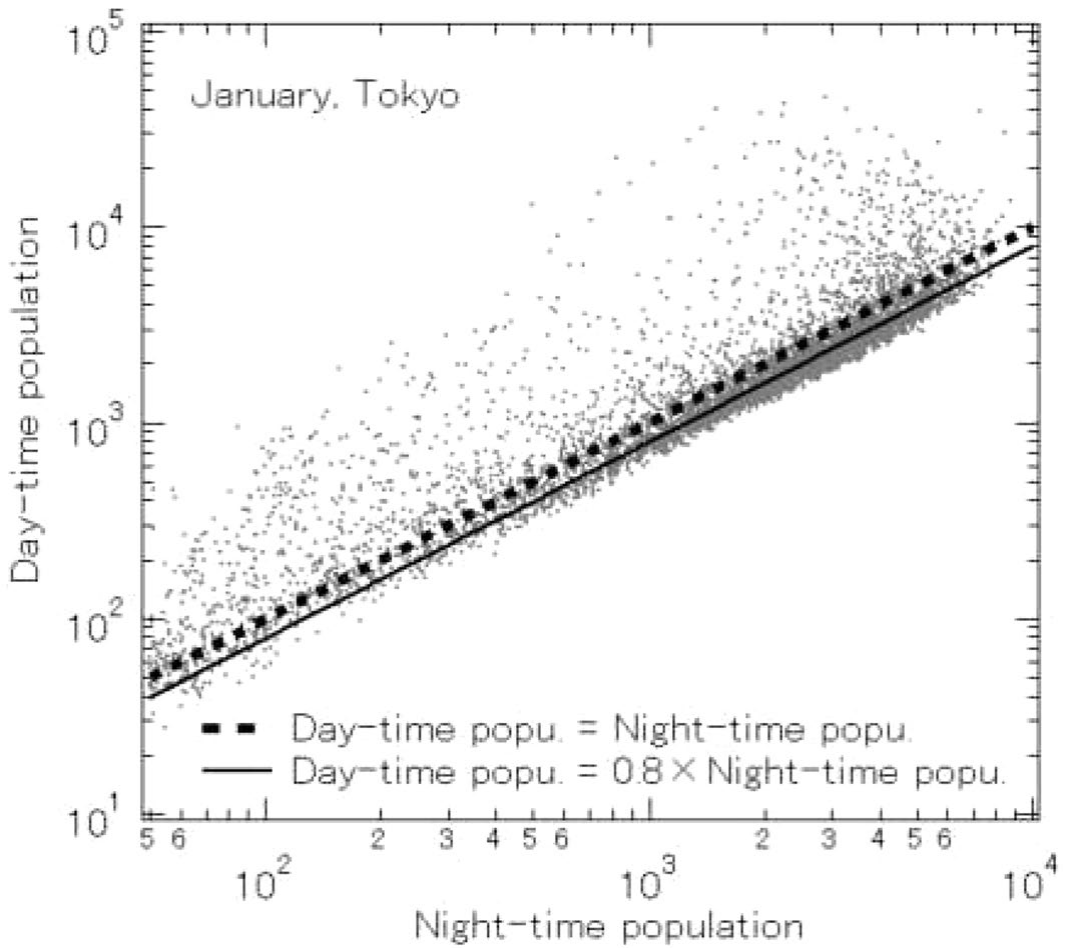
Relation between day-time and night-time populations in Tokyo on a 500 m grid for January, 2020

**Fig. 2:**
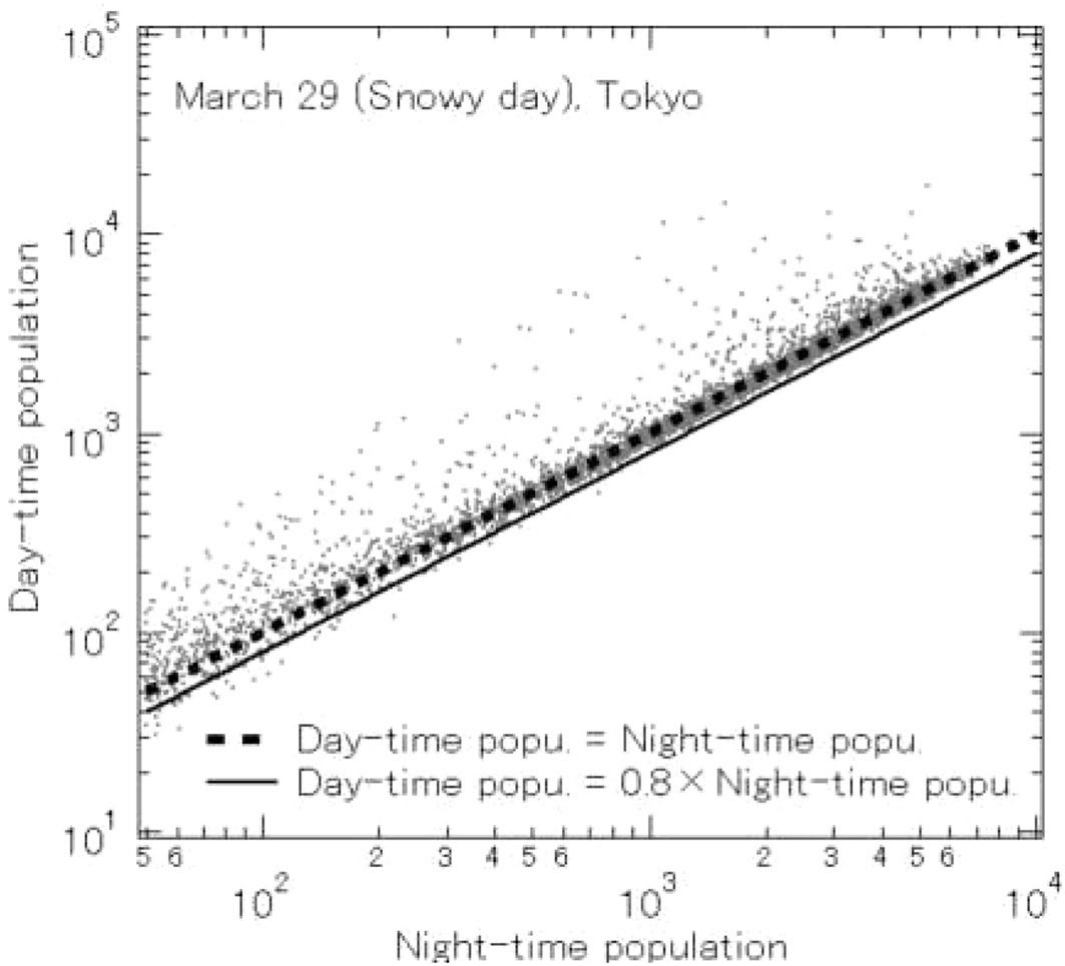
Relation between day-time and night-time population in Tokyo on a 500 m grid for March 29, 2020

This initial research was applied to find residential areas in each region. Using the population flows for the periods from midnight to 6 am and from 9 am to 6 pm on a 500 m grid, as recorded by Mobile Space Statistics, we computed the average day-time and night-time populations for Tokyo on a 500 m grid for the month of January, 2020 (from the Jan. 6 to Jan. 31). The relation between day-time and night-time populations in Tokyo on the 500 m grid for the month of January is shown in Fig. 1. The dotted line shows the case where day-time and night-time populations are equal, so that if the entire population of Tokyo stayed home during the day, the graph would perfectly match this line. In fact, as can be seen in Fig. 2, a scatter plot of day-time and night-time populations for Sunday, March 29, when there was unseasonal snow and Tokyo had issued a strong request to avoid unnecessary, non-emergency outings on weekends, most of the points are on the dotted line.

Ordinarily, many people go out during the day, so points are farther from the dotted line. Points above the dotted line indicate areas (500 m grid sections) for which day-time population is greater than night-time populations, and represent busy commercial areas like Ginza and Shinjuku, and business areas like Otemachi and Kasumigaseki. Conversely, points below the dotted line indicate areas which have day-time population less than night-time population, and represent suburban bedroom communities like the Takashimadaira housing complex and Tama New Town, and residential areas like Mitaka and Toyosu. On snow days (Fig. 2), many people refrain from going out, and there are clearly far fewer than normal points with day-time population less than 0.8 $\times$ night-time population. Thus, we can consider areas that normally have day-time population less than 0.8 $\times$ night-time population to be typical residential areas. For this research, we define points (500 m grid sections) to be residential in this way, and make observations of outflow based on this definition.

## Definition of Stay-Home Rate

Partitioning all of Japan into a 500 m-square grid, we defined each of the sections as residential if their day-time to night-time population ratios were less than 0.8 during ordinary times (taken as the average values from January 6, to January 31, 2020). The threshold of 0.8 was fixed after checking that there was not a significant difference in the results of analysis, even using thresholds of 0.9 or 0.7.

We define the number of people going out, $n_{i}(t)$, of a residential area (500 m grid section), *i*, in a certain period, *t*, as the difference between the night-time population and the day-time population, as follows

1 $$\begin{aligned} n_{i}(t)=p_{i}(t,0:00 \sim 6:00)-p_{i}(t,9:00 \sim 18:00). \end{aligned}$$

$p_{i}(t,\tau )$ is the average population for a time period, $\tau$. By aggregating the number of people going out, $n_{i}(t)$, for all residential areas in a given region, *r*, we estimate the outflow from its region,

2 $$\begin{aligned} N_{r}(t)=\sum _{i \in r}n_{i}(t). \end{aligned}$$

$N_{r}$ strongly reflects the outflow from the areas with many residents in a region *r*. For example, the contribution of $N_{r}$ in $r=$Tokyo is largest in Setagaya-ku and smallest in Aogashima-mura. For residential areas in Tokyo, the night-time population was approximately 5.3 million and the weekday daytime population was approximately 3.6 million, so the ordinary weekday outflow was about 1.7 million.

The rates of stay-home for residents of each region were calculated by comparing the outflow on each day with the standard during normal times, categorized by weekdays, Saturdays, and holidays. We illustrate the computation method with a concrete example. The outflow for residential areas in Tokyo is approximately 870,000 per hour on average on holidays from January 6 to January 31, 2020.

3 $$\begin{aligned} N_\mathrm{Tokyo}({\text {Holidays on Jan., }} 2020) \approx 870{,}000. \end{aligned}$$

The outflow on the snow day (March 29, 2020) was approximately 360,000.

4 $$\begin{aligned} N_\mathrm{Tokyo}({\text {March }} 29, \, 2020) \approx 360{,}000. \end{aligned}$$

Going out for *x* minutes is counted as *x*/60 people. Based on the outflow in January 2020, the stay-home rate on a given day, *t*, is defined as follows,

5 $$\begin{aligned} {\text {StayHomeRate}}_{r}(t)= \left\{ \begin{array}{ll} 1-\frac{N_{r}(t)}{N_{r}({\text {Weekdays on Jan., }} 2020)} &{} \text{ if } \text{ day } t \text { is a weekday}\\ 1-\frac{N_{r}(t)}{N_{r}({\text {Saturdays on Jan., }} 2020)} &{} \text{ if } \text{ day } t \text { is a Saturday}\\ 1-\frac{N_{r}(t)}{N_{r}({\text {Holidays on Jan.,}} \, 2020} &{} \text{ if } \text{ day } t \text { is a holiday} \end{array} \right. \end{aligned}$$

On the snow day, people living in Tokyo showed a stay-home rate of 59% (=0.59), relative to ordinary holiday rates. If the outflow is more significant than on a normal day, the stay-home rate will be negative. For example, on February 4, the first day of the Snow Festival in Sapporo, the stay-home rate was $-0.11$ in the Chuo-ku of Sapporo.

The rates of stay-home observe the change in the number of people out of residential areas. On the other hand, Google’s COVID-19 community mobility report observes the change in the number of people staying in residential areas based on the median value of the corresponding day of the week during the five weeks from January 3 to February 6, 2020 [19]. The definition of residential areas and the representativeness of the users who provide location information are unclear in the Mobility Report. However, the Mobility Report is widely used as a reference worldwide. We estimate the change in staying home from baseline, $C_{r}(t)$, using DOCOMO’s “Mobaku” in the same way as the stay-home rate (the change in outing), as follows:

6 $$\begin{aligned} C_{r}(t)= \left\{ \begin{array}{ll} \frac{\sum _{i \in r}p_{i}(t, 9:00 \sim 18:00)}{ \sum _{i \in r}p_{i}({\text {Weekdays on Jan.,}} \, 2020, 9:00 \sim 18:00)}-1 &{} \text{ if } \text{ day } t \text { is a weekday}\\ \frac{\sum _{i \in r}p_{i}(t, 9:00 \sim 18:00)}{ \sum _{i \in r}p_{i}({\text {Saturdays on Jan.,}} \, 2020, 9:00 \sim 18:00)}-1 &{} \text{ if } \text{ day } t \text { is a Saturday}\\ \frac{\sum _{i \in r}p_{i}(t, 9:00 \sim 18:00)}{ \sum _{i \in r}p_{i}({\text {Holidays on Jan.,}} \, 2020, 9:00 \sim 18:00)}-1 &{} \text{ if } \text{ day } t \text { is a holiday} \end{array} \right. \end{aligned}$$

**Fig. 3:**
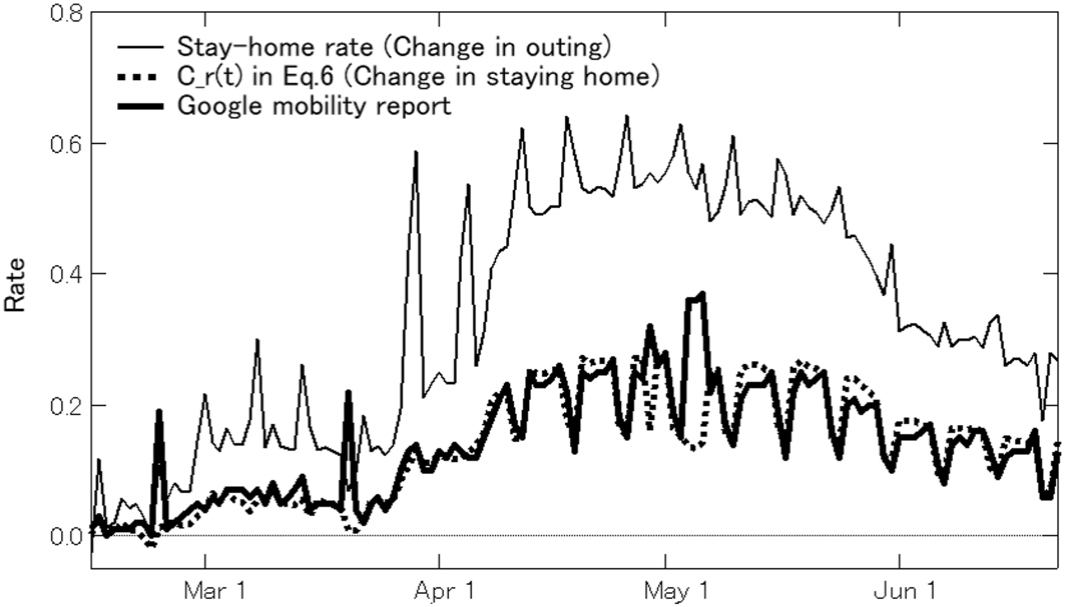
Stay-home rate (change in outing), $C_{r}(t)$ in Eq. 6 (change in staying home), and Google mobility report on residential areas in Tokyo

We compare $C_{r}(t)$ to the mobility report on residential areas and the stay-home rate. Figure 3 shows these time series in $r=$Tokyo. $C_{r}(t)$ is almost equal to the mobility report on residential areas. In other words, the residential areas we observe are not so different from those of Google, and the trend of population change in residential areas is also the same. There are divergences between $C_{r}(t)$ (is the change in staying home) and the stay-home rate (is the change in outing) because $C_{r}(t)$ and google mobility reports do not take into account the resident population (night population). The Japanese government had set a goal of reducing the number of contact opportunities (mainly going out) by 80%. To observe the achievement of the goal, the stay-home rate, which observes the number of people who go out, is useful.

## Trends in Stay-Home Compliance Throughout Japan

**Fig. 4:**
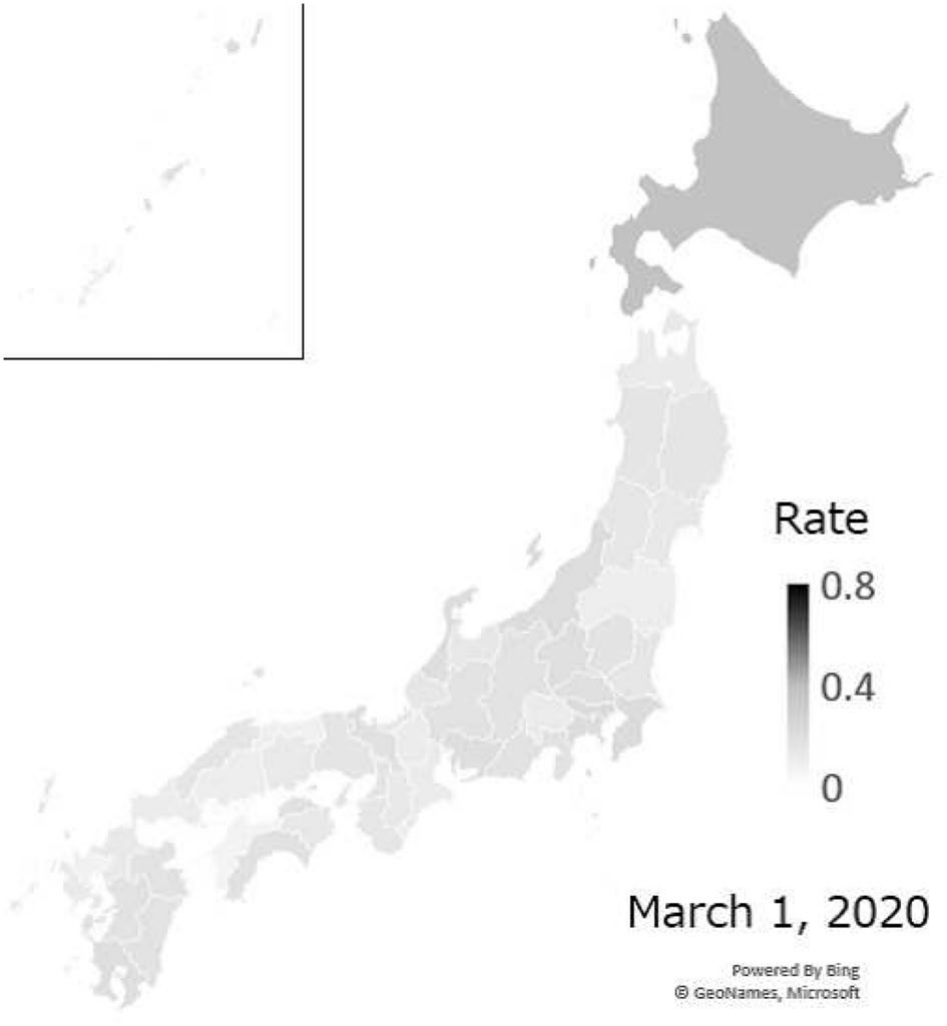
Stay-home rates on March 1, 2020 for all of Japan

**Fig. 5:**
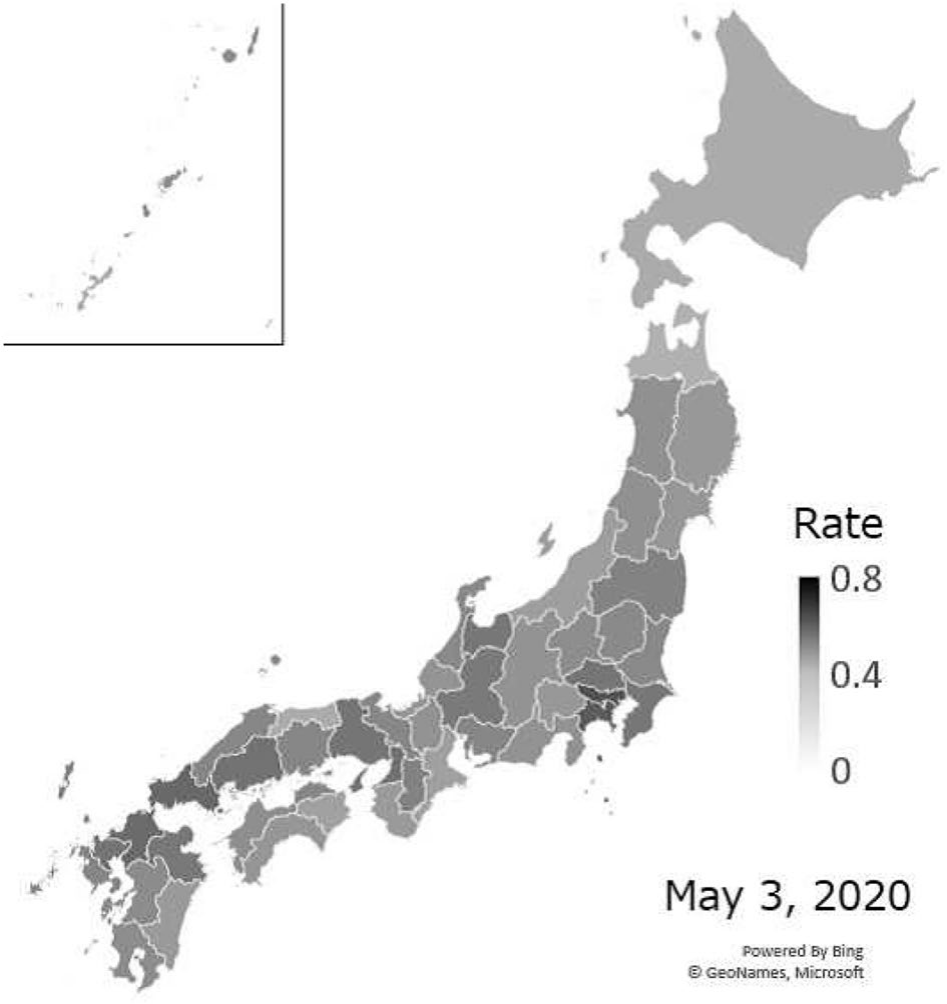
Stay-home rates on May 3, 2020, for all of Japan

**Fig. 6:**
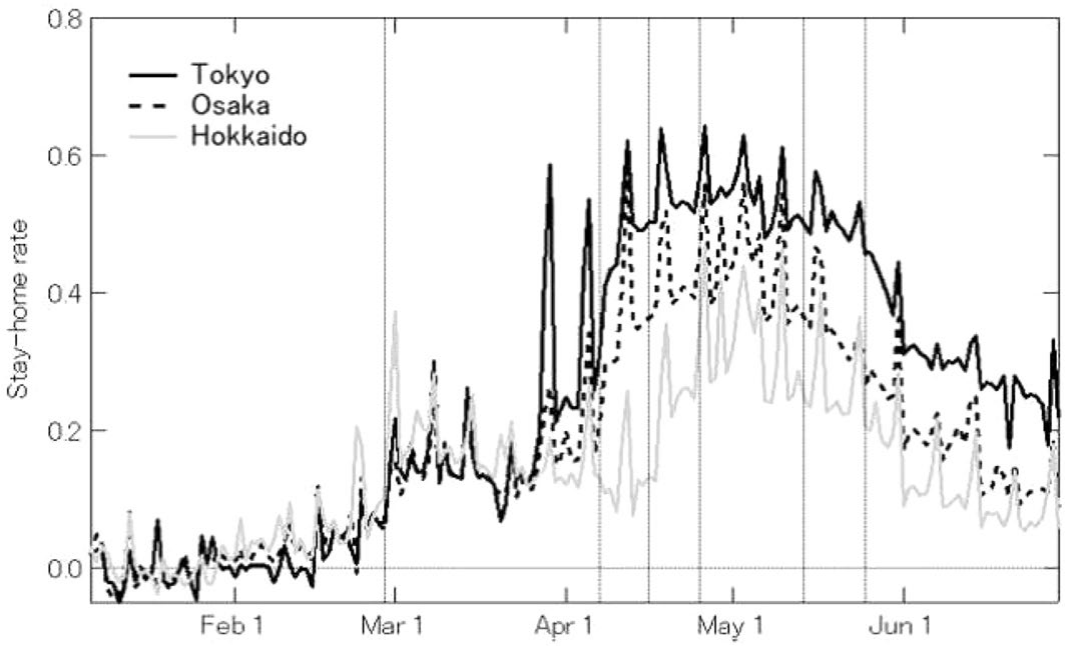
Stay-home rates in Tokyo, Osaka, and Hokkaido. Vertical lines are, from left-to right: Feb. 28 (state of emergency declared in Hokkaido), April 7 (state of emergency declared in 7 prefectures), April 16 (state of emergency declared in all prefectures), April 25 (STAY HOME week begins in Tokyo and 3 prefectures), May 14 (state of emergency lifted in 39 prefectures), May 25 (state of emergency lifted in all prefectures)

We observed residents’ stay-home compliance at the prefectural level. Fig. 4 shows a map of stay-home rates in Hokkaido on March 1, the weekend immediately following the governor’s declaration of a state of emergency. Figure 5 shows a map of all of Japan when stay-home rates peaked on May 3. Figure 6 shows the trends in Tokyo, Osaka, and Hokkaido Prefecture from January 6 to June 22, 2020.

After the declaration of a state of emergency in Hokkaido on February 28, Hokkaido led other prefectures in increasing stay-home compliance, with the rate rising to 40% on the following weekend. After that, it decreased continuously until the state of emergency was expanded to the whole country on April 16, when it began to rise again. Stay-home rates increased sharply on April 7, when a state of emergency was declared in seven prefectures, including Tokyo and Osaka. In the Tokyo area, rates peaked during the “STAY HOME" week from April 25 to May 6, and then began dropping again. At the peak, stay-home rates exceeded 60% in Tokyo, but in many prefectures, it only reached roughly 40%, and there was some variation in rates by prefecture. In prefectures with more infected people, stay-home rates were higher in proportion with the number of infected persons [20]

**Fig. 7:**
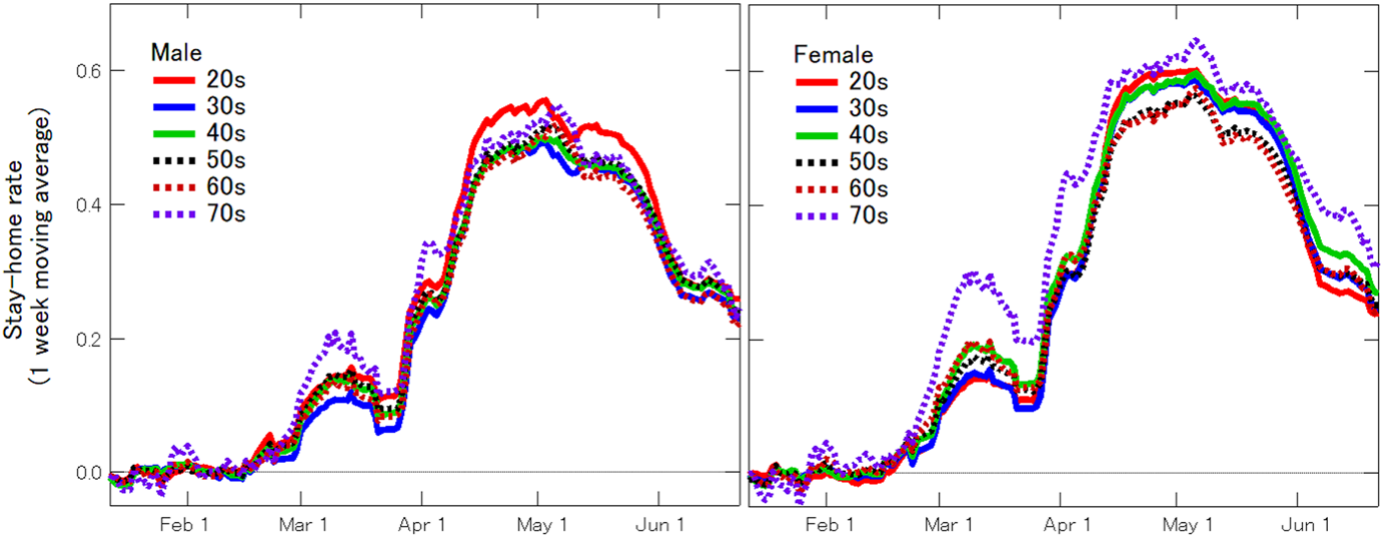
Stay-home rate for males and females in their 20s to 70s in Tokyo

Next, we show that the rates of stay-home were almost independent of sex and age. Figure 7 shows the one-week moving average of the stay-home rate for males and females in their 20s to 70s in Tokyo. During the peak period in May, the stay-home rate was slightly higher in females than in males. On the other hand, the difference by age was even more negligible. In Japan, it is often subjectively pointed out that young people do not stay-home, but young people refrain from going out at the same “rate" as other age groups. The number of young people who go out is higher than that of different age groups because the baseline of the number of people who go out is higher. In other words, young people with a high baseline needed more support to restrain themselves from going out. The government’s uniform call for all citizens to refrain from going out may have altered the retes of stay-home independent of sex and age.

## Relationship Between the Stay-Home Rate and the Spread of Infection

We now examine the relationship between stay-home compliance and the effective reproduction number, which in epidemiology, is the average expected number of infections from a single case. Here, rather than looking at stay-home rates in each region for weekdays, Saturdays and holidays, and by weekdays, we compare the average outflow per hour for each day against the standard of the average outflow per hour in January. For the effective reproduction number, we used values published by the editorial department of Toyo Keizai Online, which are computed from (positive new cases in the last 7 days/positive new cases in the prior 7 days)⌃(mean generation time/reporting interval) [21]. The mean generation time was 5 days, the reporting interval was 7 days, and the positive new case values were based on the reports for each day.

**Fig. 8:**
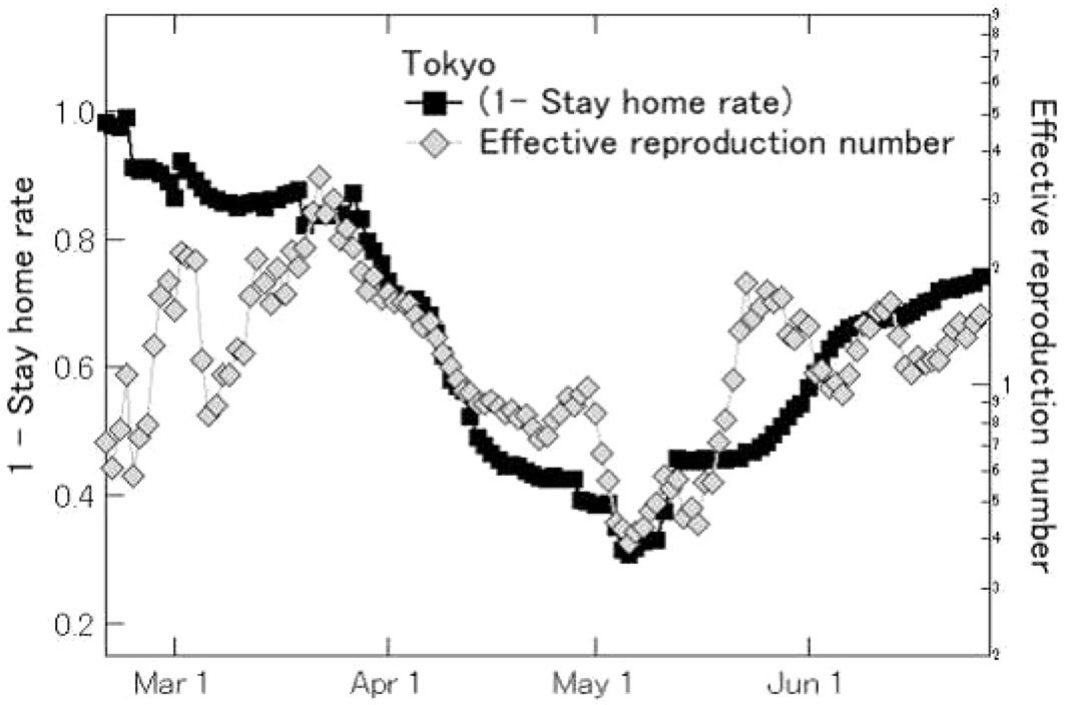
Stay-home rates and effective reproduction number for Tokyo. Stay-home rates are a moving average of the most recent 7 days. Dates for the effective reproduction numbers are 7 days prior to the day reported

Figure 8 shows the time sequence values of the effective reproduction number for Tokyo on a logarithmic scale and values for (1 − stay-home rate). The correlation coefficient between the time sequences is 0.75, showing a strong correlation. In Osaka and Hokkaido, the number of positive new cases remained low after mid-May, so the values for effective reproduction rate varied more widely during that period, but nevertheless, the strong correlation values were confirmed with values of 0.52 and 0.40 [22].

**Fig. 9:**
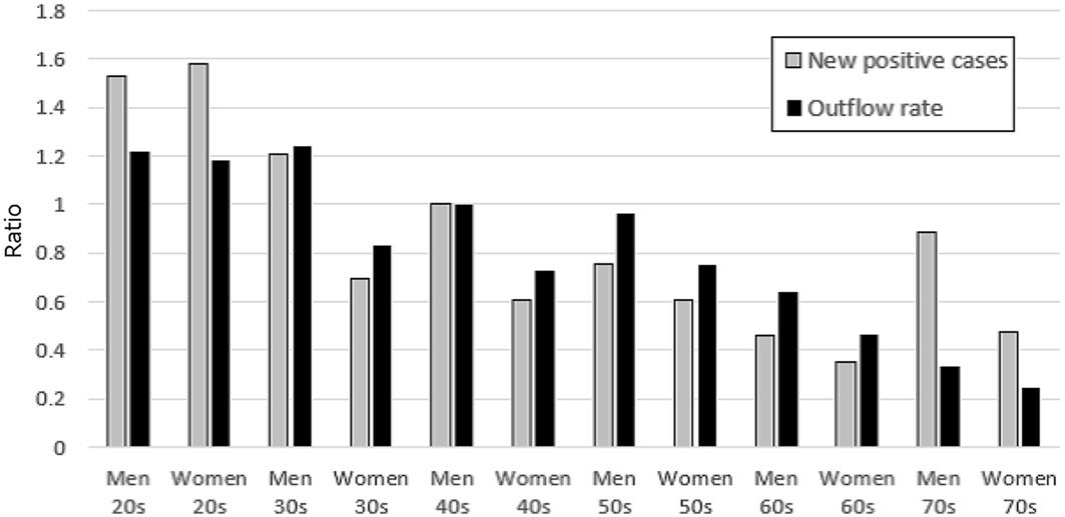
Outflow rates and new positive cases by age group and sex in Osaka City. A normalized value of 1 for males in their 40s was set for each quantity. Outflow rates from March 9 to May 3, and new positive case numbers from March 16 to May 10 were used

We also investigated the going-out rates of men and women in each age group, normalizing to a value of 1 for men in their 40s. We also studied numbers of infections by age group and sex, again normalizing to a value of 1 for men in their 40s. Figure 9 shows the results for the city of Osaka. It is clear that there were fewer infections among women 30 and older, who also go out at lower rates. As ages increased and going-out rates decreased, the number of infections similarly decreased. Similar results are obtained for other cities [22]. In other words, there exists a cross-sectional correlation between the number of new infections and the extent to which people stay at home. Note that, for those in their 70s, the number of infections tended to increase more than the going-out rate. This may be due to the effects of infection clusters in old-age facilities and hospitals.

In the first place, there are two possible reasons for the correlation between the stay-home rate and the number of new infections (or the effective reproduction number). The first is that people refrain from going out, which reduces the chance of contact with others and thus prevents infection. The second reason is that people voluntarily refrain from going out when they hear the news that the number of newly infected people is increasing, out of fear that they might become infected themselves. If the correlation is caused by the first reason, then the correlation is negative (i.e., as the stay-home rate increases, the number of new infections decreases). The direction of causality is that the increase in the stay-home rate is the cause and the decrease in the number of new infections is the result. If, on the other hand, the correlation is caused by the second reason, then the correlation is positive (i.e., an increase in the number of new infections increases the stay-home rate). The direction of causality is that the increase in the number of new infections is the cause and the increase in the stay-home rate is the result. The results observed in Figs. 8 and 9 suggest that the correlation may be caused by the first reason. However, some existing studies report that the correlation is caused by the second reason. For example, Refs. [23, 24] used the stay-home rate measured in this paper to examine the relationship with the number of new infections (or new deaths) using a panel analysis approach, and found a statistically significant positive relationship between the two variables in that when the daily number of new infections (or new deaths) increased, the daily stay-home rate also increased. This means that the correlation between the two variables is caused by the second reason. Other studies based on geolocation data in other countries also found a positive correlation between the number of new infections (or new deaths) and the extent to which people stay at home, indicating that the correlation is caused by the second reason [25].

**Fig. 10:**
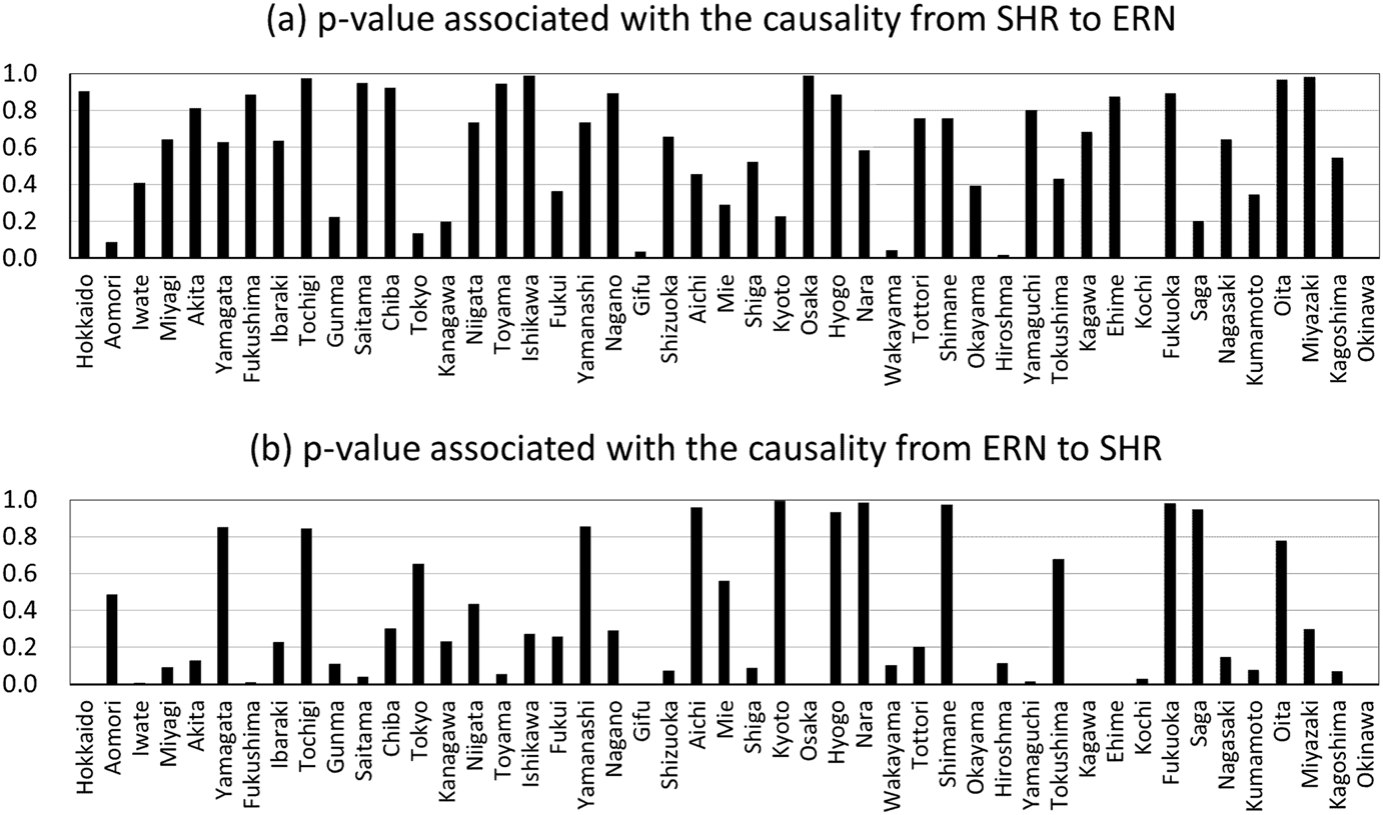
Granger causality tests between SHR (stay-home rate) and ERN (effective reproduction number)

Finally, to examine the relationship between the stay-home rate and the effective reproduction number in more detail, we perform Granger’s causality test. Specifically, we conduct a unit root test on the stay-home rate and the logarithm of the effective reproduction number for each prefecture from 1 March 2020 to 31 March 2021, and if we could not reject the null of a unit root, we take first difference before conducting Granger causality test. The results are shown in Fig. 10. Panel (a) shows the results of the causality from the stay-home rate to the effective reproduction number, expressed as *p* values associated with the tests, and (b) shows the results for the opposite causality. Panel (a) shows that the *p* value is below 5% in only five of the 47 prefectures, indicating that it cannot be said that the infection was suppressed by people refraining from going out. On the other hand, panel (b) shows that the *p* value is below 5% in 12 of the 47 prefectures, indicating that causality in this direction is also rejected in many prefectures. However, the p values are generally smaller than in panel (a), suggesting that the causality in this direction is relatively stronger than the causality in the opposite direction.

## Conclusion

In this research, we visualized the rates of stay-home for residents by region using the difference between day-time and night-time populations to detect residential areas, and then observing the numbers of people leaving residential areas (outflow). There are issues with measuring stay-home rates by observing numbers of people visiting downtown areas, such as central urban shopping centers and major train stations. The first is that we cannot eliminate the possibility that people will avoid areas being observed and go to other areas. The second is that for people visiting downtown areas, we cannot know where they reside. These issues can be resolved if we quantify the degree of stay-home using the number of people leaving residential areas (outflow). In this research, we observe outflow, so when people go to places other than central shopping areas or major train stations, such as the Enoshima tourism area, or the Togoshi Ginza shopping area, they will be reflected in the stay-home rates. For the second issue, since we are directly observing the outflow from residential areas, we know which areas have low stay-home rates. This has shown that there are significant differences in stay-home levels by region throughout Japan. Government requests for compliance were made uniformly, without regard for different regions, so they can seem exaggerated to residents in regions that are actively complying with requests, while also being inadequate for people in regions that are not incompliance. This research can be applied in future infection countermeasures because stay-home compliance is correlated with the effective reproduction number. Thus, with clear knowledge of stay-home levels on smaller regional levels, mayors and other local officials should be able to issue requests for compliance that are suited to stay-home conditions in their areas. By visualizing the effects of stay-home conditions and infection control by region, residents of each region can see whether their level of stay-home is adequate or not, and this can provide incentive toward compliance suited to the residents of the region.

In this research, we aggregated and showed stay-home rates at the prefectural level, but it is technically possible to compute stay-home rates at each residential location (500 m grid section) for the entire country. By increasing the spatial resolution, we would know in more detail, which regions have lower compliance, and it would be easier to issue compliance requests, but it could also result in discrimination among regions. If compliance of residents of a region is visualized using in this method, it will be necessary to consider the spatial resolution and how the information is published, to avoid cases of such discrimination.

The stay-home rates visualized in this research, by prefectural and municipal levels, daily values from January, 2020 to March, 2021, are published by the Mizuno Laboratory [26]. We hope that this index will help preparations to stop the spread of the disease.
